# Diagnostic Accuracy of APRI, AAR, FIB-4, FI, King, Lok, Forns, and FibroIndex Scores in Predicting the Presence of Esophageal Varices in Liver Cirrhosis: A Systematic Review and Meta-Analysis

**DOI:** 10.1097/MD.0000000000001795

**Published:** 2015-10-23

**Authors:** Han Deng, Xingshun Qi, Xiaozhong Guo

**Affiliations:** From the Liver Cirrhosis Study Group, Department of Gastroenterology, General Hospital of Shenyang Military Area, Shenyang, China (HD, XQ, XG); and Postgraduate College, Dalian Medical University, Dalian, China (HD).

## Abstract

Aspartate aminotransferase-to-platelet ratio (APRI), aspartate aminotransferase-to-alanine aminotransferase ratio (AAR), FIB-4, FI, King, Lok, Forns, and FibroIndex scores may be simple and convenient noninvasive diagnostic tests, because they are based on the regular laboratory tests and demographic data. This study aimed to systematically evaluate their diagnostic accuracy for the prediction of varices in liver cirrhosis.

All relevant papers were searched via PubMed, EMBASE, CNKI, and Wanfang databases. The area under the summary receiver operating characteristic curve (AUSROC), sensitivity, specificity, positive and negative likelihood ratio (PLR and NLR), and diagnostic odds ratio (DOR) were calculated.

Overall, 12, 4, 5, 0, 0, 4, 3, and 1 paper was identified to explore the diagnostic accuracy of APRI, AAR, FIB-4, FI, King, Lok, Forns, and FibroIndex scores, respectively. The AUSROCs of APRI, AAR, FIB-4, Lok, and Forns scores for the prediction of varices were 0.6774, 0.7275, 0.7755, 0.7885, and 0.7517, respectively; and those for the prediction of large varices were 0.7278, 0.7448, 0.7095, 0.7264, and 0.6530, respectively. The diagnostic threshold effects of FIB-4 and Forns scores for the prediction of varices were statistically significant. The sensitivities/specificities/PLRs/NLRs/DORs of APRI, AAR, and Lok scores for the prediction of varices were 0.60/0.67/1.77/0.58/3.13, 0.64/0.63/1.97/0.54/4.18, and 0.74/0.68/2.34/0.40/5.76, respectively. The sensitivities/specificities/PLRs/NLRs/DORs of APRI, AAR, FIB-4, Lok, and Forns scores for the prediction of large varices were 0.65/0.66/2.15/0.47/4.97, 0.68/0.58/2.07/0.54/3.93, 0.62/0.64/2.02/0.56/3.57, 0.78/0.63/2.09/0.37/5.55, and 0.65/0.61/1.62/0.59/2.75, respectively.

APRI, AAR, FIB-4, Lok, and Forns scores had low to moderate diagnostic accuracy in predicting the presence of varices in liver cirrhosis.

## INTRODUCTION

Variceal bleeding is one of the most lethal portal hypertension-related complications in liver cirrhosis.^1–3^ Early diagnosis and screening of varices should be warranted to improve the prognosis of liver cirrhosis. Upper gastrointestinal endoscopy is the golden diagnostic method for varices. However, given the invasiveness and relatively high cost of endoscopy and poor patients’ adherence, noninvasive diagnostic methods have been developed dramatically in the last decades.^4,5^

Recently, several systematic reviews and meta-analyses have confirmed the diagnostic performances of transient elastography (TE), spleen stiffness (SS), and platelet count to spleen diameter ratio (PSR). First, a meta-analysis of 18 studies by Shi et al^6^ found that the summary sensitivity, specificity, and area under curve (AUC) of TE for the prediction of esophageal varices (EV) were 0.87, 0.53, and 0.84, respectively; and those for the prediction of large EV were 0.86, 0.59, and 0.78, respectively. Second, a meta-analysis of 12 studies by Singh et al^7^ found that the summary sensitivity and specificity of SS for the prediction of EV were 0.78 and 0.76, respectively; and those for the prediction of large EV were 0.81 and 0.66, respectively. Third, a meta-analysis of 8 studies by Chawla et al^8^ found that the summary sensitivity and specificity of PSR for the prediction of EV were 0.89 and 0.74, respectively. Another meta-analysis of 20 studies by Ying et al^9^ also found that the summary sensitivity, specificity, and AUC of PSR for the prediction of EV were 0.92, 0.87, and 0.95, respectively. Collectively, these large meta-analyses provided the systematic evidence regarding the values of noninvasive methods for the prediction of varices. In spite of moderate to high diagnostic accuracy, they need high skills in elastography and ultrasound techniques.

By comparison, aspartate aminotransferase-to-platelet ratio (APRI), aspartate aminotransferase-to-alanine aminotransferase ratio (AAR), FIB-4, FI, King, Lok, Forns, and FibroIndex scores (Table 1), which are primarily composed of regular laboratory tests and readily available demographic data, do not need any special experiences in imaging techniques.^10–19^ They are more convenient and cheap in clinical practices. To our knowledge, the diagnostic accuracy of APRI, AAR, FIB-4, FI, King, Lok, Forns, and FibroIndex scores for the prediction of varices in liver cirrhosis have not been systematically evaluated.

**TABLE 1:**
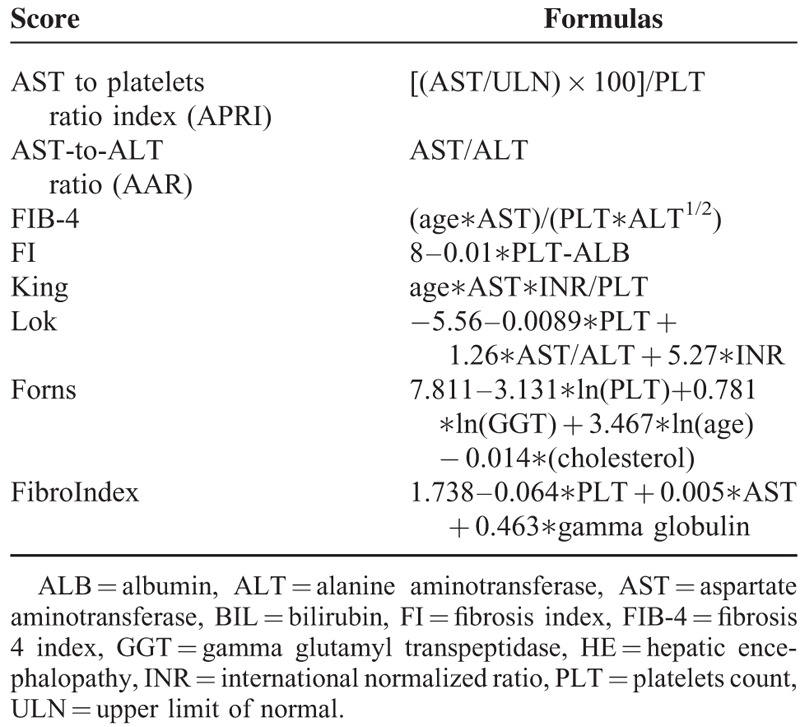
Formulas of Different Prediction Scores

## METHODS

This work is registered on PROSPERO database (registration number: CRD42015017519). Because this work is a systematic review of literatures, the ethical approval and patient consent are not necessary.

### Literature Search

All relevant papers were searched via the PubMed, EMBASE, CNKI, and Wanfang databases. PubMed and EMBASE were two major English-language databases, and CNKI and Wanfang databases were two major Chinese-language databases.

As for APRI score, the search items were as follows: **((varices) AND liver cirrhosis) AND ((APRI) OR ((aspartate aminotransferase) AND platelets))**.

As for AAR score, the search items were as follows: **((varices) AND liver cirrhosis) AND ((AAR) OR ((aspartate aminotransferase) AND alanine aminotransferase))**.

As for FIB-4 score, the search items were as follows: **((varices) AND liver cirrhosis) AND ((FIB-4 score) OR ((((aspartate aminotransferase) AND alanine aminotransferase) AND platelets) AND age))**.

As for FI score, the search items were as follows: **((varices) AND liver cirrhosis) AND ((FI score) OR ((albumin) AND platelets))**.

As for King score, the search items were as follows: **((varices) AND liver cirrhosis) AND ((King score) OR ((((aspartate aminotransferase) AND international normalized ratio) AND platelets) AND age))**.

As for Lok score, the search items were as follows: **((varices) AND liver cirrhosis) AND ((Lok) OR ((((aspartate aminotransferase) AND alanine aminotransferase) AND platelets) AND international normalized ratio))**.

As for Forns score, the search items were as follows: **((varices) AND liver cirrhosis) AND ((Forns score) OR ((((gamma-glutamyl transpeptidase) AND cholesterol) AND platelets) AND age))**.

As for FibroIndex score, the search items were as follows: **((varices) AND liver cirrhosis) AND ((FirbroIndex) OR (((platelets) AND gamma globulin) AND aspartate aminotransferase))**.

The last search was performed on April 26, 2015. Reference lists were also manually searched.

### Eligibility Criteria

*Inclusion criteria* were as follows:

1. All participants should be diagnosed with liver cirrhosis.
2. Reference tests (ie, upper gastrointestinal endoscopy) should be performed to evaluate the presence and/or grade of varices.
3. Alternative tests, such as APRI, AAR, FIB-4, FI, King, Lok, Forns, and/or FibroIndex scores, should be performed.
4. The diagnostic accuracy should be compared between reference and alternative tests.

*Exclusion criteria* were as follows:

1. Duplicates.
2. Commentaries and editorials.
3. Reviews.
4. Case reports.
5. Indexes or volumes.
6. Noncirrhotic patients.
7. Patients did not evaluate the presence of varices by upper gastrointestinal endoscopy.
8. Patients did not evaluate APRI, AAR, FIB-4, FI, King, Lok, Forns, or FibroIndex scores.
9. Diagnostic accuracy data were lacking.

### Data Extraction

The primary data were extracted as follows: first author, publication year, journal, total number of patients, age, sex, etiology of liver cirrhosis, hepatocellular carcinoma, Child–Pugh class, number of patients who underwent endoscopy, location of varices (ie, EV and/or gastric varices [GV]), prevalence of varices or large varices, definitions of large varices, cut-off value, true positive (TP) value, false positive (FP) value, false negative (FN) value, and true negative (TN) value. If the data were unclear, we contacted with the corresponding authors or submitted the letters to journal editors to validate the accuracy of data.^20–22^

### Quality Assessment

Study quality was assessed by the QUADAS (Quality Assessment of Diagnostic Accuracy Studies) score, which consisted of 14 questions assessing the risk of bias, sources of variation, and reporting quality. According to the description of every study, each item was rated as “yes” (1 point), “no” (1 point), and “unclear” (0 point). If the QUADAS score was ≥10, the study was of relatively good quality. Otherwise, the study was of poor quality.

### Data Analysis

The meta-analyses were performed by random-effects model in the Meta-DiSc software version 1.4. First, we extracted the TP, FP, FN, and TN values from original papers. If these data were missing, we recalculated these values based on the specificities and sensitivities reported.

Second, TP, FP, FN, and TN values were entered into the statistical software. The diagnostic threshold was analyzed by Spearman correlation coefficient and *P*-value. If there was no significant threshold effect, the diagnostic accuracy was evaluated by the area under the summary receiver operating characteristic curves (AUSROCs) with standard errors (SEs) and Q indexes with SEs, the summary sensitivities and specificities with 95% confidence intervals (CIs), the summary positive and negative likelihood ratios (PLRs and NLRs) with 95% CIs, and the summary diagnostic odds ratios (DORs) with 95% CIs. If there was a significant threshold effect, the diagnostic accuracy was evaluated by only AUSROCs with SEs and Q indexes with SEs, rather than sensitivities, specificities, PLRs, NLRs, or DORs.

If the AUSROC was >0.9, the diagnostic accuracy would be high; if the AUSROC was 0.7 to 0.9, the diagnostic accuracy would be moderate; if the AUSROC was 0.5 to 0.7, the diagnostic accuracy would be low. A larger Q index suggested a higher diagnostic accuracy. PLR = sensitivity/[1-specificity]. A PLR was the probability of a patient with varices testing positive divided by the probability of a patient without varices testing positive. NLR = [1-sensitivity]/specificity. An NLR was the probability of a person with varices testing negative divided by the probability of a person without varices testing negative. A PLR > 5 and an NLR < 0.2 suggested a high diagnostic accuracy. DOR = PLR/NLR. A DOR was the ratio of the odds of positivity in a patient with varices relative to the odds of positivity in a patient without varices.

Third, the heterogeneity among studies was evaluated by Chi-square test and inconsistency index. *P* < 0.1 and/or I^2^>50% was suggestive of considerable heterogeneity.

## RESULTS

### Literature Identification and Study Characteristics

#### APRI

Overall, 292 papers were identified. Among them, 12 papers were finally eligible for our study^23–34^ (Supplementary Figure 1, http://links.lww.com/MD/A468). The study characteristics are shown in Supplementary Table 1, http://links.lww.com/MD/A468. They were performed in Austria (n = 1), Brazil (n = 2), China (n = 2), England (n = 1), France (n = 1), India (n = 1), Italy (n = 2), Japan (n = 1), and Romania (n = 1). Two and 10 of them were published in abstracts and full-texts, respectively. Both EV and GV were evaluated in one paper, and EV alone was evaluated in 11 papers. Prevalence of varices was 26% to 72.6%. Prevalence of large varices was 3.3% to 48.2%. QUADAS score was 8 to 12 (Supplementary Table 2, http://links.lww.com/MD/A468). Seven and 5 of them were of relatively good and poor quality, respectively.

#### AAR

Overall, 241 papers were identified. Among them, 4 papers were finally eligible for our study^23,24,28,30^ (Supplementary Figure 2, http://links.lww.com/MD/A468). The study characteristics are shown in Supplementary Table 3, http://links.lww.com/MD/A468. They were performed in England (n = 1), France (n = 1), and Italy (n = 2). One and 3 of them were published in abstracts and full-texts, respectively. EV alone was evaluated in all of the 4 included papers. Prevalence of varices was 26% to 56.9%. Prevalence of large varices was 13.5% to 27.1%. QUADAS score was 8 to 12 (Supplementary Table 4, http://links.lww.com/MD/A468). Two and 2 of them were of relatively good and poor quality, respectively.

#### FIB-4 Score

Overall, 17 papers were identified. Among them, 5 papers were finally eligible for our study^26,28,30,31,35^ (Supplementary Figure 3, http://links.lww.com/MD/A468). The study characteristics are shown in Supplementary Table 5, http://links.lww.com/MD/A468. They were performed in Egypt (n = 1), England (n = 1), Italy (n = 1), Japan (n = 1), and Romania (n = 1). One and 4 of them were published in abstracts and full-texts, respectively. EV alone was evaluated in all of the 5 included papers. Prevalence of varices was 26% to 76.9%. Prevalence of large varices was 13.5% to 49.2%. QUADAS score was 7–12 (Supplementary Table 6, http://links.lww.com/MD/A468). Four and 1 of them were of relatively good and poor quality, respectively.

#### FI Score

Overall, 52 papers were identified. Among them, no papers were finally eligible for our study (Supplementary Figure 4, http://links.lww.com/MD/A468).

#### King Score

Overall, 4 papers were identified. Among them, no papers were finally eligible for our study (Supplementary Figure 5, http://links.lww.com/MD/A468).

#### Lok Score

Overall, 26 papers were identified. Among them, 4 papers were finally eligible for our study^24,30,31,35^ (Supplementary Figure 6, http://links.lww.com/MD/A468). The study characteristics are shown in Supplementary Table 7, http://links.lww.com/MD/A468. They were performed in Egypt (n = 1), Romania (n = 1), Italy (n = 1) and France (n = 1). All of them were published in full-texts. EV alone was evaluated in all of the 4 included papers. Prevalence of varices was 35.7% to 76.9%. Prevalence of large varices was 18.6% to 49.2%. QUADAS score was 9 to 12 (Supplementary Table 8, http://links.lww.com/MD/A468). Three and 1 of them were of relatively good and poor quality, respectively.

#### Forns Score

Overall, 22 papers were identified. Among them, 3 papers were finally eligible for our study^30,31,35^ (Supplementary Figure 7, http://links.lww.com/MD/A468). The study characteristics are shown in Supplementary Table 9, http://links.lww.com/MD/A468. They were performed in Egypt (n = 1), Romania (n = 1), and Italy (n = 1). All of them were published in full-texts. EV alone was evaluated in all of the 3 included papers. Prevalence of varices was 56.9% to 76.9%. Prevalence of large varices was 19% to 49.2%. QUADAS score was 10 to 12 (Supplementary Table 10, http://links.lww.com/MD/A468). All of them were of relatively good quality.

#### FibroIndex

Overall, 15 papers were identified. Among them, only 1 paper was finally eligible for our study^30^ (Supplementary Figure 8, http://links.lww.com/MD/A468). The study characteristics are shown in Supplementary Table 11, http://links.lww.com/MD/A468. It was performed in Italy, and was published in full-texts. EV alone was evaluated. Prevalence of varices was 56.9%. Prevalence of large varices was 19%. QUADAS score was 12 (Supplementary Table 12, http://links.lww.com/MD/A468).

### Meta-Analyses

The results of meta-analyses are shown in Table 2. Notably, while FIB-4 and Forns scores were employed to predict the presence of varices, there were significant diagnostic threshold effects. Thus, the sensitivities, specificities, PLRs, NLRs, and DORs of FIB-4 and Forns scores for the prediction of varices in liver cirrhosis were not calculated.

**TABLE 2:**
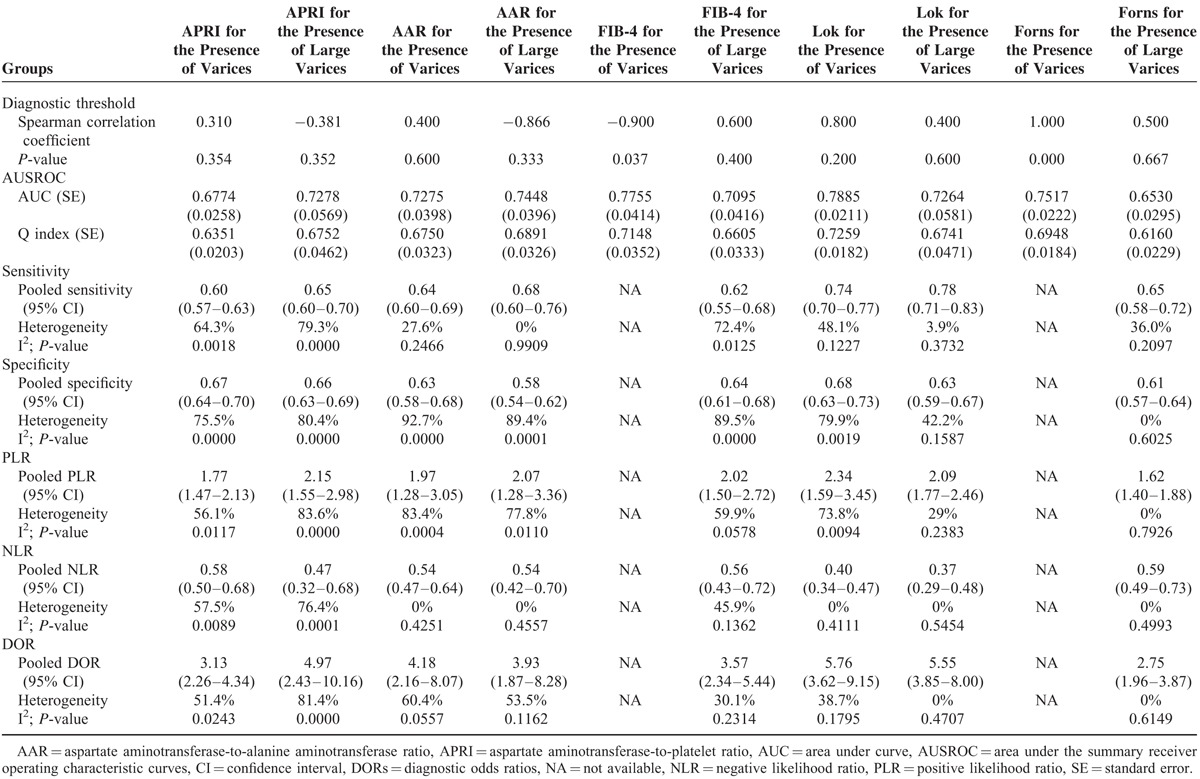
Results of Meta-Analyses: An Overview

#### SROC

**FIGURE 1:**
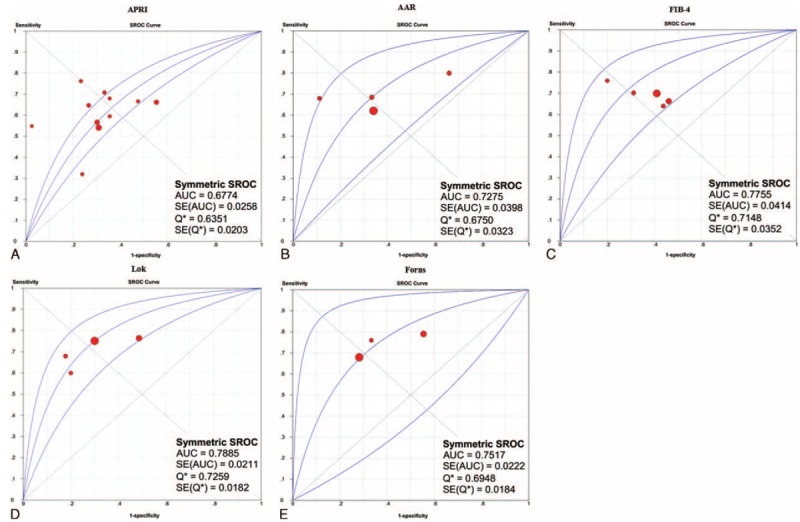
SROCs of APRI, AAR, FIB-4, Lok, and Forns scores for the prediction of varices in liver cirrhosis. Panel A: APRI; panel B: AAR; panel C: FIB-4; panel D: Lok; panel E: Forns.

The SROCs of APRI, AAR, FIB-4, Lok, and Forns scores for the prediction of varices are shown in Figure 1. Lok score had the largest summary AUC followed by FIB-4, Forns, AAR, and APRI scores.

**FIGURE 2:**
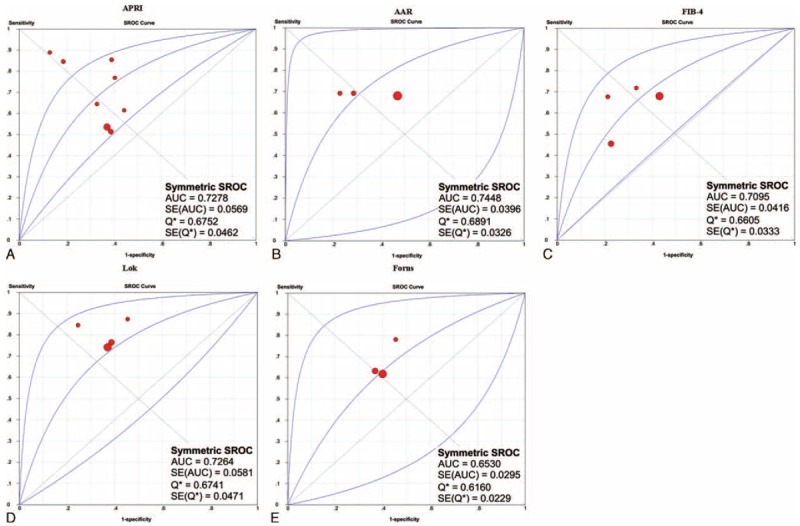
SROCs of APRI, AAR, FIB-4, Lok, and Forns scores for the prediction of large varices in liver cirrhosis. Panel A: APRI; panel B: AAR; panel C: FIB-4; panel D: Lok; panel E: Forns.

The SROCs of APRI, AAR, FIB-4, Lok, and Forns scores for the prediction of large varices are shown in Figure 2. AAR score had the largest summary AUC followed by APRI, Lok, FIB-4, and Forns scores.

#### Sensitivity

**FIGURE 3:**
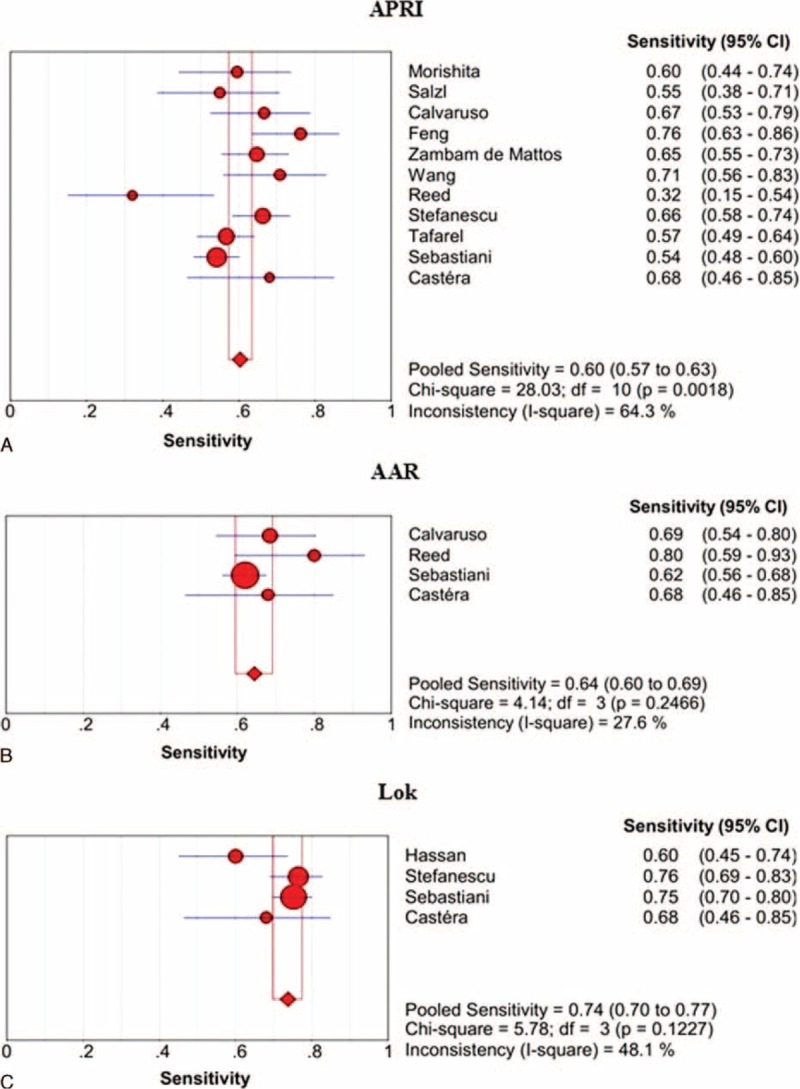
Summary sensitivities of APRI, AAR, and Lok scores for the prediction of varices in liver cirrhosis. Panel A: APRI; panel B: AAR; panel C: Lok.

The sensitivities of APRI, AAR, and Lok scores in the prediction of varices are shown in Figure 3. Lok score had the largest summary sensitivity followed by AAR and APRI scores. The lower limit of 95% CI of Lok score was larger than the upper limits of 95% CIs of AAR and APRI scores.

**FIGURE 4:**
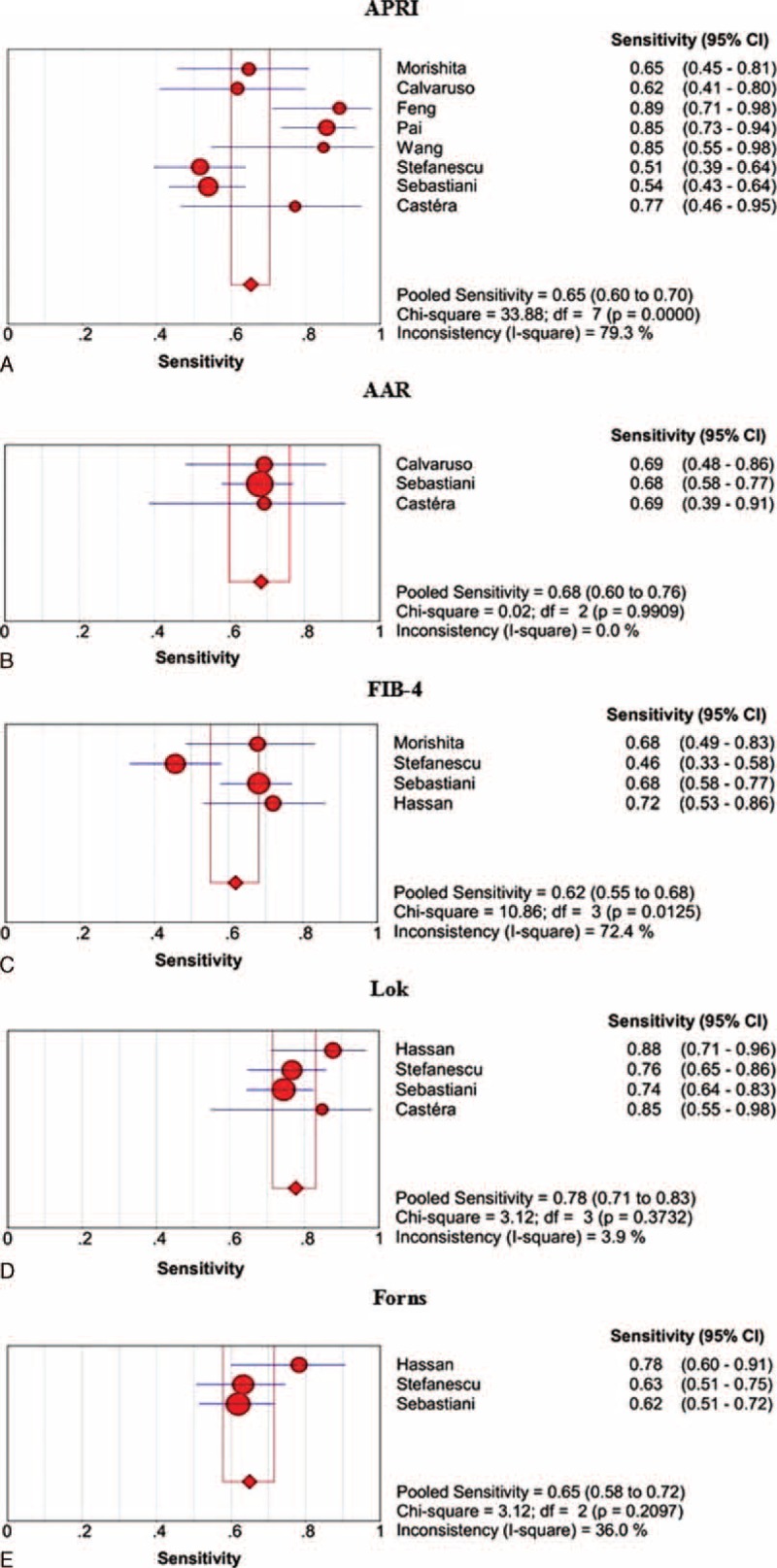
Summary sensitivities of APRI, AAR, FIB-4, Lok, and Forns scores for the prediction of large varices in liver cirrhosis. Panel A: APRI; panel B: AAR; panel C: FIB-4; panel D: Lok; panel E: Forns.

The sensitivities of APRI, AAR, FIB-4, Lok, and Forns scores in the prediction of large varices are shown in Figure 4. Lok score had the largest summary sensitivity followed by AAR, Forns, APRI, and FIB-4 score, but their 95% CIs were overlapping.

#### Specificity

**FIGURE 5:**
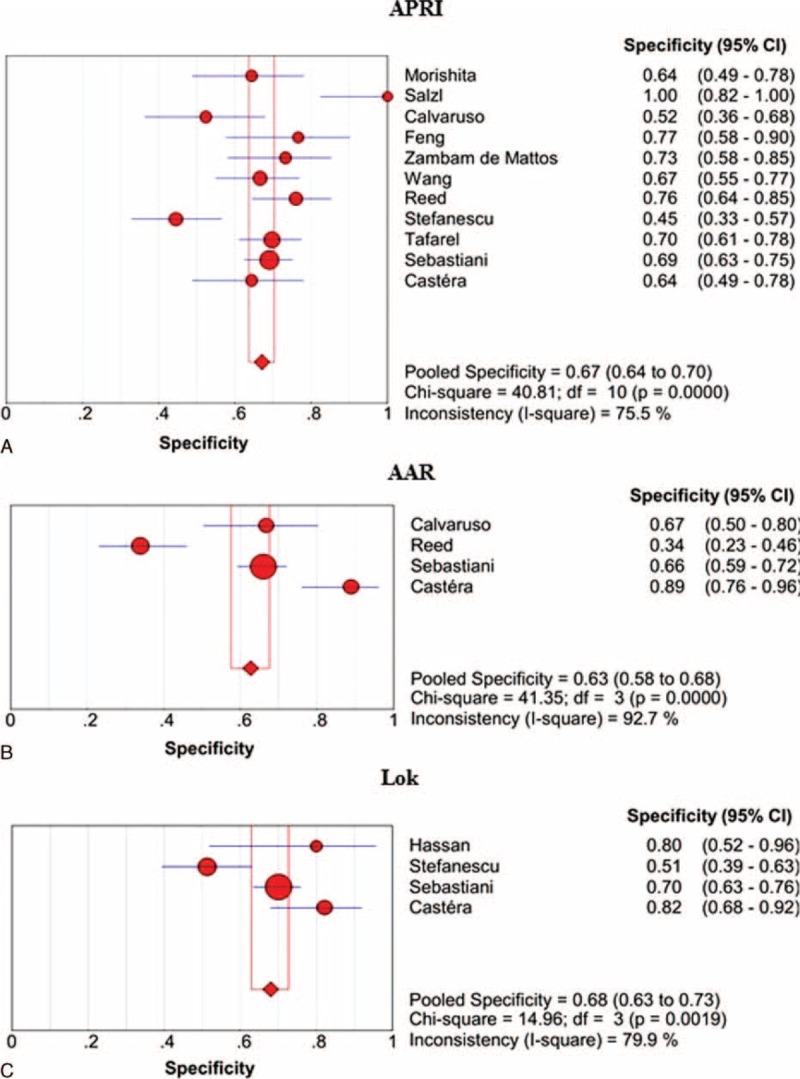
Summary specificities of APRI, AAR, and Lok scores for the prediction of varices in liver cirrhosis. Panel A: APRI; panel B: AAR; panel C: Lok.

The specificities of APRI, AAR, and Lok scores in the prediction of varices are shown in Figure 5. Lok score had the largest summary sensitivity followed by APRI and AAR scores, but their 95% CIs were overlapping.

**FIGURE 6:**
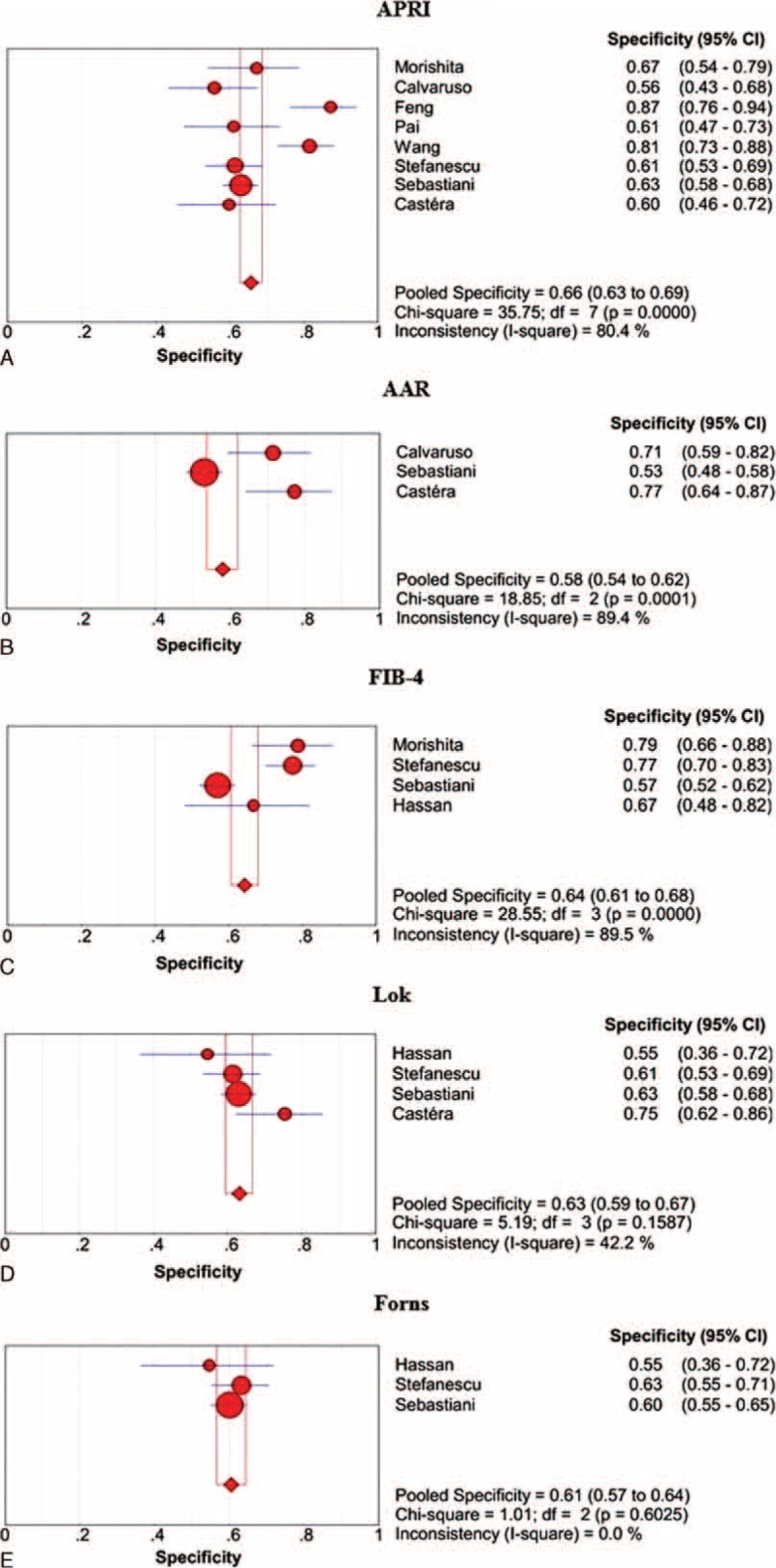
Summary specificities of APRI, AAR, FIB-4, Lok, and Forns scores for the prediction of large varices in liver cirrhosis. Panel A: APRI; panel B: AAR; panel C: FIB-4; panel D: Lok; panel E: Forns.

The specificities of APRI, AAR, FIB-4, Lok, and Forns scores in the prediction of large varices are shown in Figure 6. APRI score had the largest summary specificity followed by FIB-4, Lok, Forns, and AAR scores, but their 95% CIs were overlapping.

#### PLR

The PLRs of APRI, AAR, and Lok scores in the prediction of varices are shown in Supplementary Figure 9, http://links.lww.com/MD/A468. Lok score had the largest summary PLR followed by AAR and APRI scores, but their 95% CIs were overlapping.

The PLRs of APRI, AAR, FIB-4, Lok, and Forns scores in the prediction of large varices are shown in Supplementary Figure 10, http://links.lww.com/MD/A468. APRI score had the largest summary PLRs followed by Lok, AAR, FIB-4, and Forns score, but their 95% CIs were overlapping.

#### NLR

The NLRs of APRI, AAR, and Lok scores in the prediction of varices are shown in Supplementary Figure 11, http://links.lww.com/MD/A468. Lok score had the smallest summary NLR followed by AAR and APRI scores, but their 95% CIs were overlapping.

The NLRs of APRI, AAR, FIB-4, Lok, and Forns scores in the prediction of large varices are shown in Supplementary Figure 12, http://links.lww.com/MD/A468. Lok score had the smallest summary NLR followed by APRI, AAR, FIB-4, and Forns scores, but their 95% CIs were overlapping.

#### DOR

The DORs of APRI, AAR, and Lok scores in the prediction of varices are shown in Supplementary Figure 13, http://links.lww.com/MD/A468. Lok score had the largest summary DOR followed by AAR and APRI scores, but their 95% CIs were overlapping.

The DORs of APRI, AAR, FIB-4, Lok, and Forns scores in the prediction of large varices are shown in Supplementary Figure 14, http://links.lww.com/MD/A468. Lok score had the largest summary DOR followed by APRI, AAR, FIB-4, and Forns scores, but their 95% CIs were overlapping.

## DISCUSSION

Currently, noninvasive diagnosis of varices is an important topic in the management of liver cirrhosis. However, our recent questionnaire survey suggested that noninvasive diagnostic tests of varices in liver cirrhosis were rarely used in clinical practices.^36^ This might be primarily because their reliability was questioned. We performed a systematic review to identify the papers regarding the role of APRI, AAR, FIB-4, FI, King, Lok, Forns, and FibroIndex scores for the prediction of varices in liver cirrhosis. Notably, to maximize the number of papers retrieved, a total of 4 databases were systematically searched, the reference lists were manually searched, and the publication language and date were not limited. Additionally, we contacted with the authors to validate the accuracy of relevant data. Generally, as we evaluated the diagnostic accuracy for the prediction of varices in liver cirrhosis, the meta-analyses demonstrated the following: the AUROCs ranged from 0.6774 to 0.7885, the summary sensitivities ranged from 0.60 to 0.74, the summary specificities ranged from 0.63 to 0.68, the summary PLRs ranged from 1.77 to 2.34, the summary NLRs ranged from 0.40 to 0.58, and the summary DORs ranged from 3.13 to 5.76. As we evaluated the diagnostic accuracy for the prediction of large varices, the meta-analysis demonstrated the following: the AUROCs ranged from 0.653 to 0.7448, the summary sensitivities ranged from 0.62 to 0.78, the summary specificities ranged from 0.58 to 0.66, the summary PLRs ranged from 1.62 to 2.15, the summary NLRs ranged from 0.37 to 0.59, and the summary DORs ranged from 2.75 to 5.55. These findings suggested low to moderate diagnostic accuracy of APRI, AAR, FIB-4, Lok, and Forns scores in predicting the presence of varices or large varices in liver cirrhosis. According to several previous meta-analyses,^6–9^ we had to acknowledge that APRI, AAR, FIB-4, Lok, and Forns scores might be inferior to TE, SS, and PSR for the prediction of varices. Thus, their clinical utility might be compromised.

APRI, AAR, FIB-4, Lok, and Forns scores were firstly introduced to evaluate the presence and severity of liver fibrosis in patients with chronic viral hepatitis. Their components include age, alanine aminotransferase (ALT), aspartate aminotransferase (AST), cholesterol, gamma glutamyl transpeptidase (GGT), international normalized ratio (INR), and platelets (PLT). In 2002, Forns et al^18^ identified age, GGT, cholesterol, and PLT as independent predictors of fibrosis in patients with chronic hepatitis C. Forns score = 7.811 − 3.131 × ln(PLT) + 0.781 × ln(GGT) + 3.467 × ln(age) − 0.014 × (cholesterol). In 2003, Wai et al^10^ constructed the APRI score to predict both significant fibrosis and cirrhosis in patients with chronic hepatitis C. APRI score = [(AST/upper limit of normal range) × 100]/PLT. At the same year, Giannini et al^11^ also evaluated the correlation of AAR score with the histological stage and prognosis of hepatitis C virus-related liver diseases. AAR score = AST/ALT. In 2005, Lok et al^17^ developed a predictive model of liver cirrhosis in patients with chronic hepatitis C. Lok score was composed of PLT, AST, ALT, and INR. In 2006, Sterling et al^12^ developed the FIB-4 score to predict liver fibrosis in patients with HIV/HCV coinfection. FIB-4 score = (age^∗^AST)/(PLT^∗^ALT^1/2^). In 2007, Vallet-Pichard et al^13^ further confirmed that FIB-4 score was simple, accurate, and inexpensive for the assessment of liver fibrosis in patients with hepatitis C.

Since their original development, numerous studies have confirmed the values of APRI, AAR, FIB-4, Lok, and Forns scores in the diagnosis of liver fibrosis and cirrhosis. Recently, 2 meta-analyses by Jin et al^37^ and Shaheen and Myers^38^ explored the diagnostic accuracy of APRI for the prediction of hepatitis B and C-related fibrosis, respectively. More recently, a meta-analysis by Xiao et al^39^ suggested that APRI and FIB-4 should have moderate sensitivity and specificity of detecting the presence of liver fibrosis. The mean AUCs of APRI and FIB-4 for the prediction of significant fibrosis were 0.72 and 0.76, respectively. Similarly, our meta-analysis also demonstrated that the summary AUCs of APRI, AAR, FIB-4, Lok, and Forns scores for the prediction of varices were 0.6774, 0.7275, 0.7755, 0.7885, and 0.7517, respectively; and those for the prediction of large varices were 0.7278, 0.7448, 0.7095, 0.7264, and, 0.6530, respectively. The diagnostic accuracy appeared to be similar among them, because their 95% CIs of sensitivities, specificities, PLRs, NLRs, and DORs were mostly overlapping.

However, we had to acknowledge that their sensitivities and specificities were relatively low (about 70%). Thus, the reliability of these noninvasive diagnostic methods of varices in liver cirrhosis should be questioned.

Our systematic review and meta-analysis had several limitations. First, although 8 scores were systematically reviewed in our study, we did not identify any relevant papers to explore the role of FI or King score for the prediction of varices. FI score was based on platelets and albumin.^14^ King score consisted of age, AST, INR, and platelets.^15^ Additionally, the diagnostic accuracy of FibroIndex was evaluated in only one paper. FibroIndex was composed of platelet count, AST, and gamma globulin.^19^ All of them played important roles in diagnosing the liver fibrosis. However, their associations with varices in liver cirrhosis needed to be explored. Second, the diagnostic threshold effect was statistically significant in the meta-analyses regarding the role of FIB-4 and Forns scores in predicting the presence of varices in liver cirrhosis. Thus, their sensitivities, specificities, PLRs, NLRs, and DORs were not combined. Third, the heterogeneity among studies was statistically significant in most of meta-analyses. Certainly, we employed random-effects models to perform all meta-analyses.

In conclusion, based on a systematic review and meta-analysis, APRI, AAR, FIB-4, Lok, and Forns scores had low to moderate diagnostic accuracy in predicting the presence of varices and large varices in liver cirrhosis. Thus, they might not be adequate to replace the use of upper gastrointestinal endoscopy. Further studies should be warranted to confirm these findings and to explore the potential correlations of FI, King, and FibroIndex scores with the presence of varices in liver cirrhosis.
