# Diagnostic Accuracy of APRI, AAR, FIB-4, FI, King, Lok, Forns, and FibroIndex Scores in Predicting the Presence of Esophageal Varices in Liver Cirrhosis: A Systematic Review and Meta-Analysis: Erratum

**DOI:** 10.1097/01.md.0000480464.27661.5b

**Published:** 2016-02-08

In the article “Diagnostic Accuracy of APRI, AAR, FIB-4, FI, King, Lok, Forns, and FibroIndex Scores in Predicting the Presence of Esophageal Varices in Liver Cirrhosis: A Systematic Review and Meta-Analysis”,^1^ which appeared in Volume 94, Issue 42 of *Medicine*, the “no” item rating in the Quality Assessment section was originally incorrectly labeled as “1”. The “no” item rating actually has a value of “0”. The article has since been corrected online.
